# Albuminuria, Cerebrovascular Disease and Cortical Atrophy: among Cognitively Normal Elderly Individuals

**DOI:** 10.1038/srep20692

**Published:** 2016-02-15

**Authors:** Eun Bin Cho, Hee-Young Shin, Sang Eon Park, Phillip Chun, Hye Ryoun Jang, Jin-ju Yang, Hee Jin Kim, Yeo Jin Kim, Na-Yeon Jung, Jin San Lee, Juyoun Lee, Young Kyoung Jang, Eun Young Jang, Mira Kang, Jong-Min Lee, Changsoo Kim, Ju-Hong Min, Seungho Ryu, Duk L. Na, Sang Won Seo

**Affiliations:** 1 Department of Neurology, Samsung Medical Center, Sungkyunkwan University School of Medicine; 2 Neuroscience Center, Samsung Medical Center, Seoul, Korea; 3 Department of Neurology, Gyeongsang National University Changwon Hospital, Gyeongsang National University School of Medicine, Changwon, Korea; 4 Center for Health Promotion, Samsung Medical Center, Sungkyunkwan University School of Medicine, Seoul, Korea; 5 Samsung Advanced Institute for Health Sciences and Technology, Sungkyunkwan University, Seoul, Korea; 6 Department of Emergency Medicine Behavioral Emergencies Research Lab, San Diego, CA, USA; 7 Department of Biology, University of California San Diego, CA, USA; 8 Department of Medicine, Samsung Medical Center, Sungkyunkwan University School of Medicine, Seoul, Korea; 9 Department of Biomedical Engineering, Hanyang University, Seoul, Korea; 10 Department of Neurology, Pusan National University Hospital, Pusan National University College of Medicine and Biomedical Research Institute, Busan, Korea; 11 Department of Preventive Medicine and the Institute for Environmental Research, Yonsei University College of Medicine, Seoul, Korea; 12 Divison of Preventive Medicine, Department of Medicine, Brigham and Women’s Hospital and Harvard Medical School, Boston, MA; 13 Department of Occupational and Environmental Medicine, Kangbuk Samsung Hospital, Sungkyunkwan University School of Medicine, Seoul, Korea; 14 Department of Health Sciences and Technology, SAIHST, Sungkyunkwan University, Seoul, Korea; 15 Department of Clinical Research Design & Evaluation, SAIHST, Sungkyunkwan University, Seoul, Korea

## Abstract

We tested the hypothesis that decreased glomerular filtration rate and albuminuria have different roles in brain structure alterations. We enrolled 1,215 cognitively normal individuals, all of whom underwent high-resolution T1-weighted volumetric magnetic resonance imaging scans. The cerebral small vessel disease burdens were assessed with white matter hyperintensities (WMH), lacunes, and microbleeds. Subjects were considered to have an abnormally elevated urine albumin creatinine ratio if the value was ≥17 mg/g for men and ≥25 mg/g for women. Albuminuria, but not estimated glomerular filtration rate (eGFR), was associated with increased WMH burdens (p = 0.002). The data was analyzed after adjusting for age, sex, education, history of hypertension, diabetes mellitus, hyperlipidemia, ischemic heart disease, stroke, total cholesterol level, body mass index, status of smoking and alcohol drinking, and intracranial volume. Albuminuria was also associated with cortical thinning, predominantly in the frontal and occipital regions (both p < 0.01) in multiple linear regression analysis. However, eGFR was not associated with cortical thickness. Furthermore, path analysis for cortical thickness showed that albuminuria was associated with frontal thinning partially mediated by WMH burdens. The assessment of albuminuria is needed to improve our ability to identify individuals with high risk for cognitive impairments, and further institute appropriate preventive measures.

There is increasing evidence that even in earlier stages, chronic kidney disease (CKD) is associated with the increased risk of cognitive impairments or development of dementia^1^,^2^. A strong candidate mechanism for their associations might be related to the hemodynamic similarities between the vascular beds of the kidney and the brain. In fact, some studies have shown that individuals with CKD have more cerebral small vessel disease (CSVD) burdens including white matter hyperintensities (WMH) and lacunes^3^,^4^,^5^,^6^. However, independent of CSVD, several studies also revealed the link between CKD and brain atrophy suggesting other potential mechanisms, such as neurodegenerative hypothesis^7^,^8^.

Consistent with other chronic medical conditions which gradually developed over time, the recent guidelines for the definition and classification of CKD made it gain recognition as a worldwide public health problem which is emphasized for prevention, early detection, and management. The early stages of CKD are defined based on the combination of decreased kidney function, quantified as glomerular filtration rate (GFR), and the degree of glomerular damage, most often represented by albuminuria^9^. In addition to the core role of GFR in the pathophysiological complications, albuminuria may also be an early sign of glomerular disease leading to CKD. Previous studies have shown that albuminuria and reduced estimated GFR (eGFR) are independent risk factors for cardiovascular events or earlier mortality, suggesting that albuminuria and reduced eGFR may be markers of different pathologic processes^10^,^11^. Therefore, the pattern of association with CSVD or brain atrophy might differ between decreased eGFR and albuminuria.

In the present study, we examined the associations between CKD, represented by eGFR or albuminuria, and increased CSVD burdens or cortical thinning in a large sample of cognitively normal individuals. Cortical thickness is an important biomarker for predicting cognitive impairment in cognitively normal individuals, since cortical thinning precedes the onset of cognitive decline^12^,^13^,^14^. Therefore, our study objective was to test how decreased eGFR and albuminuria had different roles in brain structure alteration including CSVD and cortical thinning. We hypothesized that albuminuria has a more direct association with brain structure alteration than decreased eGFR does because albuminuria is associated with endothelial dysfunction which might have a critical role in changing brain structures^15^,^16^. To test our hypotheses, we investigated the effects of decreased GFR or albuminuria on CSVD or cortical thickness. Second, we also evaluated the relationships between CKD markers, CSVD burdens, and cortical thickness using path analyses.

## Materials and Methods

The methods were carried out in accordance with the approved guidelines.

### Participants

A total of 1,589 participants were enrolled in this study. They visited the Samsung Medical Health Promotion Center between September 2008 and March 2013 for disease-preventive medical check-ups including dementia and underwent detailed medical examinations including estimated GFR (eGFR) and urine albumin to creatinine ratio (UACR). As a part of the comprehensive exam, participants could opt for a neurological and neurocognitive screening package that included a brain magnetic resonance imaging (MRI). Out of them, we excluded the following number of participants from this study: 21 participants who were under 45 years of age; 154 participants with significant cognitive impairment defined by scores below the 16th percentile in age-, gender-, and education-matched norms according to the Mini-Mental Status Exam (MMSE) or through an interview conducted by a qualified neurologist; and 171 participants with missing data on demographics. We also excluded 28 participants with unreliable analyses of cortical thickness due to head motion, blurring of MRI, inadequate registration to a standardized stereotaxic space, misclassification of tissue type, and inexact surface extraction. Finally, a total of 1215 participants were included in this study.

This study was approved by the Institutional Review Board of Samsung Medical Center. The requirement for participant’s consent was waived since we used retrospective de-identified data collected during health exam visits.

### Baseline assessment

The examinations were conducted by trained personnel according to standard protocol. The typical health screening program practiced at our center includes height, weight, a complete blood cell count, basic chemistry, serologic test, blood coagulation test, thyroid function test, assay for tumor markers, stool/urine analysis, abdominal ultrasonography, gastrofiberscopy, chest radiography, pulmonary function test, and electrocardiography. All have been previously described^17^. Quality-control procedures were performed in accordance with the Korean Association of Laboratory Quality Control. Medical information was gathered through questionnaires, which included the questions on physician diagnosed diseases, medication history, cigarette smoking and alcohol consumption. We categorized educational attainment into three groups: 0–9 years, 10–14 years (high school graduate to college graduate), and 15 or more years. Diabetes mellitus was defined as a history of taking diabetes medication or a fasting blood sugar level ≥126 mg/dl. Hypertension was defined as a history of hypertension medication use, systolic blood pressure (SBP) ≥140 mmHg or diastolic blood pressure (DBP) ≥90 mmHg measured by an electronic sphygmomanometer after participants were in a relaxed state for more than five minutes. Participants were classified as non-smokers, ex-smokers, or current smokers. Alcohol consumption was used to categorize subjects as non-drinkers or current drinkers.

Kidney function was evaluated by eGFR using the simplified Modification of Diet in Renal Disease Study equation that is defined as the eGFR (ml/min/1.73 m^2^) = 86.3 × (serum creatinine)^−1.154^ × age^−0.203^, from which the result is multiplied by 0.742 for females^18^. Serum creatinine was measured using a modified kinetic Jaffe reaction. And, UACR was measured by a spot collection of morning urine, of which was obtained during a fasting state. Subjects were considered to have an abnormally elevated UACR (above microalbuminuria level) if the value was ≥17 mg/g for men and ≥25 mg/g for women^19^,^20^.

### Acquisition of MRI data

All participants underwent a 3D volumetric brain MRI scan. An Achieva 3.0-Tesla MRI scanner (Philips, Best, the Netherlands) was used to acquire 3D T1 Turbo Field Echo (TFE) MRI data using a sagittal slice thickness of 1.0 mm, overcontiguous slices with 50% overlap and no gap, a repetition time of 9.9 ms, an echo time of 4.6 ms, a flip angle of 8°, and a matrix size of 240 × 240 pixels reconstructed to 480 × 480 over a field of view of 240 mm. Radiologists inspected all MRI data for evidence of brain tumors of any kind, major infarctions (except lacunar infarctions), and hemorrhages (observed as low intensity areas in T2-weighted images).

### WMH visual rating scale

Whitematter hyperintensities (WMH) visual rating scale was modified using the Fezekas scale. On this scale, periventricular WMH (PWMH) were classified as P1 (cap and band <5 mm), P2 (5 mm ≤ cap or band <10 mm), and P3 (10 mm ≤ cap or band); deep WMH (DWMH) was classified into D1 (maximum diameter of deep white matter lesion <10 mm), D2 (10 mm ≤ lesion < 25 mm), and D3 (≥25 mm). The inter-rater reliability of the WMH visual rating scale was presented in the previous study, which was proven to be a great support to our current study (intraclass correlation coefficient between 0.726 and 0.905)^21^. Another previous study showed that WMH visual rating scale correlated with automated measured volume of WMH^22^.

The results were combined to give a final ischemia classification of minimal, moderate, or severe. The combinations of D1 with P1 (D1P1) and D1 with P2 (D1P2) were classified as ‘minimal’. The combinations D2P1, D3P1, D2P2, D3P2, D1P3, and D2P3 were classified as ‘moderate’, while D3P3 was classified as ‘severe’. A previous study showed that this ischemia classification system distinguished the presence of vascular risk factors and the severity of cerebrovasculare disease (CVD) markers.

### Number of lacunes

The lacune was defined as a small lesion (≤15 mm in diameter) with low signal on T1-, high signal on T2-weighted images, and perilesional halo on FLAIR images. Two experienced neurologists who were blinded to the patients’ data reviewed the number and location of the lacunes on 20 axial slices of FLAIR. The rate of agreement between these two neurologists was 83.0% and consensus was reached in any case of discrepancy.

### Number of microbleeds

Cerebral microbleeds were defined according to the criteria proposed by Greenberg *et al.*, ranging <10 mm in diameter^23^. Two experienced neurologists, who were blinded to patients’ data, reviewed the number and location of cerebral microbleeds on 20 axial slices of T2 FFE-MRI. The rate of agreement between these two neurologists was 92.3% and a consensus was reached in any case of discrepancy.

### Image processing for cortical thickness measurement

Images were processed by the standard Montreal Neurological Institute anatomic pipeline. This processing includes stereotaxic space transformation^24^, intensity normalization^25^, tissue segmentation^26^, and automatic surface extraction of the inner and outer cortex^27^. Cortical thickness was measured in native space due to limitations of linear stereotaxic normalization, as previously described in detail^28^. As MRI volumes were transformed and cortical surface models were extracted in stereotaxic space, we reconstructed cortical surfaces by applying inverse transformation^29^ and calculated cortical thickness in native space using Euclidean distance between the linked vertices of the inner and outer surfaces^27^. We included intracranial volume (ICV) as a covariate to control the brain size effect in statistical analyses. ICV was calculated by measuring the volume within the brain mask which was generated using the FSL brain extraction tool algorithm^30^.

### Nonlinear registration of cortical surface

To compare the thicknesses of corresponding regions among the subjects, the vertices of each subject were nonlinearly registered onto a surface group template using surface-based two-dimensional registration with a sphere-to-sphere warping algorithm^31^.

Using the transformation, an individual cortical surface took the lobar labels from the template on which lobar boundaries were previously defined^28^. For the global analysis, average values of the thickness of the whole vertex in each hemisphere and lobar region were used. Diffusion smoothing with a full-width half-maximum of 20 mm was used to blur each map of the cortical thickness, which increased the signal-to-noise ratio and statistical power^27^,^29^.

### Statistical analyses

We divided the patients into five groups according to eGFR: <60 (GFR1), 60–74 (GFR2), 75–89 (GFR3), 90–104 (GFR4), and ≥105 (GFR5) ml/min/1.73 m^2^. The categories with eGFR 90-104 ml/min/1.73 m^2^ (GFR4) and normal UACR were used as references, respectively. Baseline characteristics were compared between participants in a reference category and other categories. Regarding continuous variables, further comparison was done using Student’s *t*-test and analysis of variance (ANOVA). While for categorical variables, Chi-square test was used for comparison.

We first evaluated the independent associations of eGFR and albuminuria as categorical variables with CSVD burdens (WMH, lacunes or microbleeds) and regional cortical thickness. For the analysis, WMH were dichotomized into absent/minimal WMH or moderate to severe WMH. Also, the numbers of lacune and microbleed were dichotomized into categories of being absent or present. Logistic and liner regression analyses were performed after adjusting for age, sex, history of hypertension, diabetes mellitus, hyperlipidemia, ischemic heart disease, and stroke, fasting glucose, systolic blood pressure, diastolic blood pressure, total cholesterol level, body mass index, status of current smoking and alcohol drinking, education level and intracranial volume. P values were corrected by Bonferroni’s method due to multiple testing and p < 0.05 was considered statistically significant. All these analyses were executed using SPSS version 20.

To analyze the localized statistical map of cortical thickness on the surface model related to albuminuria, the elevated UACR was entered as the predictor and cortical thickness on a vertex-by-vertex; which then we performed a multiple linear regression analysis. This was also carried out after controlling the same confounders as the above analysis. The resulting statistical maps were thresholded, using the false discovery rate (FDR) theory at a Q value of 0.05, after pooling the *p* values from the regression analysis.

To evaluate whether CSVD burdens mediates the effect of albuminuria on cortical thickness, path analyses were performed after controlling for age, sex, history of hypertension, diabetes mellitus, hyperlipidemia, ischemic heart disease, and stroke, fasting glucose, systolic blood pressure, diastolic blood pressure, total cholesterol level, body mass index, status of current smoking and alcohol drinking, education level and intracranial volume.

## Results

The clinical characteristics of the participants are summarized in Table 1. Compared to the normal UACR group, elevated UACR group had the following characteristics: older age, greater number of males and current alcohol drinkers, higher levels of fasting glucose, greater BMI and increased vascular risk factors such as hypertension, diabetes mellitus, and hyperlipidemia. GFR1 group (the lowest eGFR) was older and had more frequent hypertension, higher levels of BMI, and lower levels of total cholesterol than the GFR4 group (reference group). The proportion of subjects with elevated UACR was higher in the GFR1 group compared to the GFR4 group.

### CKD markers and CSVD burdens

Elevated UACR group had more frequent moderate to severe WMH compared to the normal UACR group (OR, 2.3; 95% CI, 1.4-3.8; p = 0.002) (Table 2). However, there were no differences in CSVD burdens between the GFR4 group and other GFR groups.

### CKD markers and cortical thickness

Elevated UACR group showed significant cortical thinning in the frontal and occipital regions, of which were results obtained after controlling possible confounders (Table 3). There were no differences in cortical thickness between the GFR4 group and other GFR groups.

**Figure 1:**
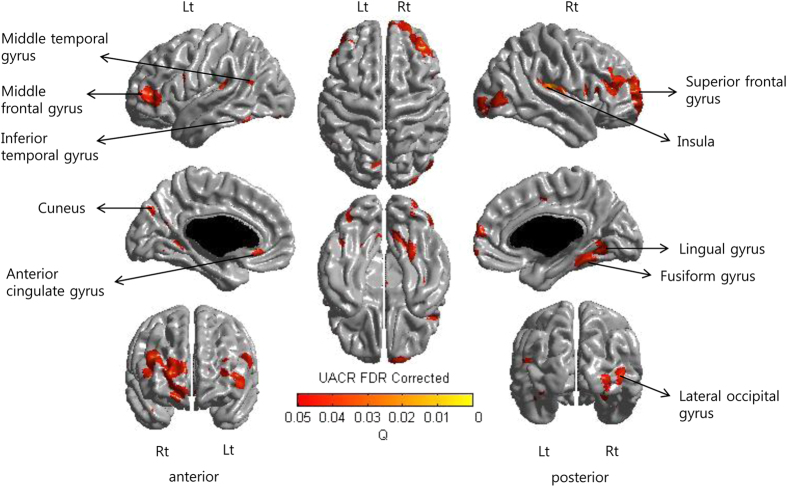
Statistical map of cortical thinning related to albuminuria. The subjects with albuminuria showed cortical thinning predominantly in bilateral middle frontal, insula, and lingual gyri; right superior frontal, lateral occipital, and fusiform gyri; and left cuneus, anterior cingulate, and middle and inferior temporal gyri.

Topography of cortical thinning showed that elevated UACR group had cortical thinning in the bilateral middle frontal, insula, and lingual gyri; right superior frontal, lateral occipital, and fusiform gyri; and left cuneus, anterior cingulate, and middle and inferior temporal gyri (Fig. 1).

### Path analysis

**Figure 2:**
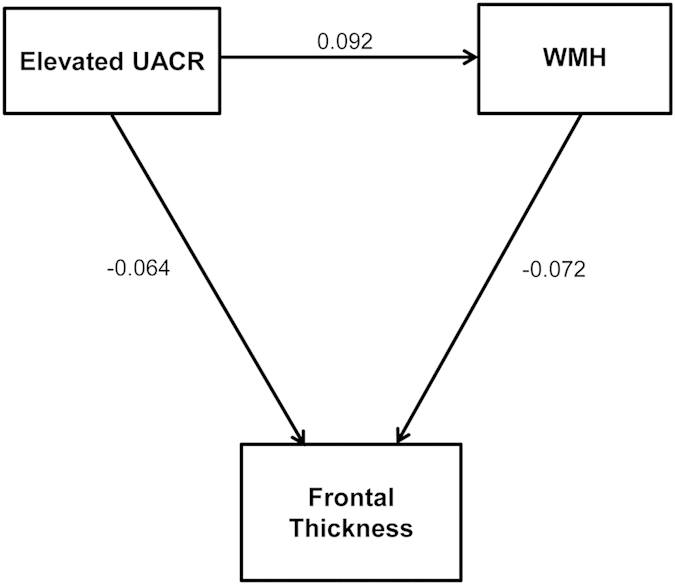
Schematic diagram of the path analyses for frontal cortical thickness. Elevated urine albumin to creatinine ratio (UACR) was associated with moderate to severe white matter hyperintensities (WMH), which were further associated with frontal cortical thinning. Elevated UACR was also associated with frontal thinning without being mediated by WMH burdens. Albuminuria was entered as a predictor and WMH volume was entered as a mediator. Age, sex, history of hypertension, diabetes mellitus, hyperlipidemia, ischemic heart disease, and stroke, fasting glucose, systolic blood pressure, diastolic blood pressure, total cholesterol level, body mass index, status of current smoking and alcohol drinking, education level and intracranial volume were entered as covariates. Numbers on the paths are standardized coefficients that were statistically significant.

The path analysis for frontal cortical thickness showed goodness of fit to the data: Chi-square =59.470, degrees of freedom = 46, p = 0.088, comparative fit index = 0.995 and root-mean-square error of approximation =0.016 (Fig. 2). Elevated UACR was associated with moderate to severe WMH, which were further associated with frontal cortical thinning. Elevated UACR was also associated with frontal thinning without being mediated by WMH burdens.

## Discussion

This study reports novel findings about the relationship between CKD markers, CSVD burdens, and cortical thickness in a large cognitively normal cohort. Our findings suggested that assessment of albuminuria is needed to improve our ability in identifying high risk individuals for cognitive impairments, so that we can institute appropriate preventive measures.

Our conclusion that albuminuria was associated with cortical thinning with or without the mediation of WMH burden is supported by the following observations: (1) albuminuria was associated with increased WMH burdens; (2) albuminuria also contributed to cortical thinning, predominantly in the frontal and occipital regions; (3) path analyses for cortical thinning showed that albuminuria was associated with frontal thinning with or without the mediation of WMH burdens. Although previous studies evaluated the relationships between kidney function and imaging biomarkers^5^,^6^,^7^,^8^, the concurrent relationships between kidney function, CSVD, and cortical thickness remains to be fully established.

The mechanisms by which albuminuria was associated with cortical thinning with the mediation of WMH burden might be explained by shared pathophysiology between kidney and brain. It has been suggested that albuminuria may simply represent the renal manifestations of systemic endothelial dysfunction^16^,^32^. That is, gradual endothelial damage and leakage of serum proteins into the brain’s interstitial spaces could lead to increased WMH burdens^6^. In fact, pathological studies revealed that enlarged perivascular spaces and perivascular demyelination were observed in WMH on MRI^33^. Considering previous studies showing that WMH were associated with cortical thinning through secondary degeneration after subcortical injury, it might, therefore, be reasonable to explain that albuminuria affect WMH, which in turn lead to cortical thinning^15^,^34^. In fact, the topography of cortical thinning related to albuminuria generally overlapped with that related to WMH^15^,^34^.

We also found that albuminuria affected cortical thinning without the mediation of WMH. Their pathobiology remains unclear. However, it might be related to several explanations that albuminuria affect cortical thinning through changes at the microvascular level, which, however, are too subtle to be detected by conventional MRI. In fact, these microvascular changes including cortical microinfarct, apoptosis, and microstructural abnormalities in normal appearing white matter correlate with cognitive impairments^35^,^36^,^37^. Therefore, the pathogenesis of cortical changes related to albuminuria might include cortical microinfarct, apoptosis, and/or microstructural changes, as well as changes resulting in WMHs. Alternatively, other vascular risk factors such as hyperhomocysteinemia, hemostatic abnormalities, oxidative stress and inflammation may mediate the association between albuminuria and cortical thinning^38^. Finally, it is possible that albuminuria is associated with the production of uremic toxins such as guanidine compounds, which causes directly neuronal toxicity and eventually resulting in cortical thinning^2^.

However, unlike previous studies, we did not find any relationship of eGFR with CSVD markers or cortical thinning after accounting for potential confounding factors. Several studies have shown that lower eGFR levels were associated with greater white matter lesions in the elderly^3^,^4^,^39^. Other studies also revealed that decreased eGFR levels were associated with decreased cortical thickness^7^,^8^. Especially, a previous study from our group showed that Alzheimer’s disease dementia (AD) patients with decreased eGFR levels had cortical thinning than those with normal eGFR levels^40^. These discrepancies might be explained by differences in the study population. In our study, participants are cognitively normal and most of them (94.7%) had normal to mildly reduced kidney function, whereas those in the previous studies had AD or severe degree of kidney dysfunction. By contrast, the presence of albuminuria had a significant association with WMH burdens or brain atrophy in these participants in line with the previous studies^7^,^41^. Therefore, our findings suggested that endothelial dysfunction represented with albuminuria might have a critical role in developing CSVD or thinning in the brain. Further studies should be needed to investigate the effects of endothelial cell dysfunction on the brain related to albuminuria.

Some methodological issues need to be considered. First, this study is a cross-sectional study. Therefore, these findings do not allow for causal inference regarding the directionality of the relationship. Second, in the present study, CSVD markers were assessed as “none” to “presence” because of non-normalized distribution, which may not properly reflect the effects of eGFR abnormalities or albuminuria. Third, the duration or etiologies of kidney dysfunction were not available, which could partly explain the changes in brain structure. Finally, our participants were recruited from attendees to a comprehensive preventive health exam not covered by national medical insurance, which may limit the generalizability of this study to the general population. However, the strengths of this study is analyzing cortical thickness data measured by sophisticated methods in a large sample size, which allows for precise estimations of the effects of albuminuria on brain structures.

## Additional Information

**How to cite this article**: Cho, E. B. *et al.* Albuminuria, Cerebrovascular Disease and Cortical Atrophy: among Cognitively Normal Elderly Individuals. *Sci. Rep.* **6**, 20692; doi: 10.1038/srep20692 (2016).

## Figures and Tables

**Table 1:** Characteristics of study participants.

N = 1,215	eGFR (ml/min/1.73 m^2^)					UACR (mg/g)	
	GFR1 (<60)	GFR2 (60–74)	GFR3 (75–89)	GFR3 (90–104, reference))	GFR5 (>105)	Normal UACR (reference)	Elevated UACR
Numbers	65 (5.3)	272 (22.4)	463 (38.1)	325 (26.7)	90 (7.4)	1092 (89.9)	123 (10.1)
Elevated UACR	16 (24.6)^*^	29 (10.7)	40 (8.6)	24 (7.4)	14 (15.6)^*^	0 (0)	100 (100)
Age, years	69.6 ± 8.2^*^	65.6 ± 7.5^*^	63.2 ± 7.0	62.2 ± 7.7	61.8 ± 7.9	63.5 ± 7.6	65.8 ± 7.4^*^
Sex: female	23 (35.4)^*^	131 (48.2)	265 (57.2)	170 (52.3)	62 (68.9)^*^	595 (56.4)	34 (29.3)^*^
Education							
<10 years	15 (23.1)	69 (25.4)	119 (25.7)	93 (28.6)	27 (30.0)	282 (26.7)	31 (26.7)
10~14 years	17 (26.2)	73 (26.8)	153 (33.0)	107 (32.9)	36 (40.0)	341 (32.3)	36 (31.0)
≥15 years	33 (50.8)	130 (47.8)	191 (41.3)	125 (38.5)	27 (30.0)	432 (40.9)	49 (42.2)
Hypertension	47 (72.3)^*^	143 (52.6)^*^	189 (40.8)	118 (36.3)	45 (50.0)	446 (42.3)	76 (65.5)^*^
Diabetes	17 (26.2)	45 (16.5)	62 (13.4)	47 (14.5)	14 (15.6)	143 (13.6)	33 (28.4)^*^
Hyperlipidemia	15 (23.1)	68 (25.0)	114 (24.6)	79 (24.3)	23 (25.6)	251 (23.8)	40 (34.5)^*^
Coronary heart disease	7 (10.8)	22 (8.1)	24 (5.2)	21 (6.5)	3 (3.3)	64 (6.1)	12 (10.3)
Stroke	5 (7.7)	5 (1.8)	23 (5.0)	8 (2.5)	4 (4.4)	37 (3.5)	6 (5.2)
Smoking							
Non-smoker	31 (47.7)	166 (61.0)	302 (65.2)	209 (64.3)	64 (71.1)	791 (65.5)	56 (48.3)^*^
Ex-smoker	28 (43.1)	83 (30.5)	128 (27.6)	89 (27.4)	21 (23.3)	286 (27.1)	50 (43.1)^*^
Current smoker	6 (9.2)	23 (8.5)	33 (7.1)	27 (8.3)	5 (5.6)	78 (7.4)	10 (8.6)
Alcohol							
No	28 (43.1)	128 (47.1)	237 (51.2)	151 (46.5)	49 (54.4)	527 (50.0)	44 (37.9)^*^
Yes	37 (56.9)	144 (52.9)	226 (48.8)	174 (53.5)	41 (45.6)	528 (50.0)	72 (62.1)^*^
Fasting glucose	101.4 ± 20.5	99.6 ± 22.6	99.0 ± 19.5	98.5 ± 18.5	100.9 ± 26.0	98.2 ± 18.8	109.0 ± 30.6^*^
Cholesterol	178.3 ± 36.0^*^	192.8 ± 35.3	196.0 ± 37.7	194.9 ± 35.6	195.2 ± 40.7	194.6 ± 36.5	188.5 ± 40.2
Body mass index	25.0 ± 3.6^*^	24.1 ± 2.8	23.7 ± 2.7	23.7 ± 3.3	23.7 ± 3.0	23.8 ± 2.9	24.4 ± 3.6^*^
SBP (mmHg)	125.2 ± 19.4	125.1 ± 19.7	124.9 ± 17.9	123.1 ± 16.8	125.9 ± 19.3	124.2 ± 17.9	128.6 ± 19.7^*^
DBP (mmHg)	74.2 ± 11.9	75.1 ± 10.8	74.9 ± 10.5	73.3 ± 10.4	73.7 ± 10.1	74.1 ± 10.5	76.2 ± 11.3^*^
WMH							
Moderate to severe	17 (26.2)^*^	45 (16.5)	65 (14.0)	39 (12.0)	12 (13.3)	144 (13.2)	34 (27.7)^*^
Lacunes	0.5 ± 1.5^*^	0.2 ± 0.5	0.2 ± 0.6	0.2 ± 0.7	0.2 ± 0.6	0.2 ± 0.6	0.4 ± 1.1^*^
Yes	12 (18.5)	37 (13.6)	44 (9.5)	33 (10.2)	9 (10.0)	109 (10.0)	26 (21.1)^*^
Microbleeds	0.7 ± 2.9	0.1 ± 0.5	0.2 ± 1.4	0.2 ± 1.9	0.2 ± 0.5	0.2 ± 1.4	0.5 ± 2.2^*^
Yes	9 (13.8)	27 (9.9)	38 (8.2)	30 (9.2)	10 (11.1)	96 (8.8)	18 (14.6)^*^
ICV (mm^3^)	1372441.7 ± 142026.1	1359796.9 ± 124610.9	1368566.2 ± 118300.2	1380767.3 ± 123177.2	1364610.1 ± 117243.3	1365263.1 ± 120085.2	1409890.9 ± 134926.2^*^
Cortical thickness (mm)							
Overall	2.99 ± 0.15^*^	3.03 ± 0.11^*^	3.06 ± 0.10	3.06 ± 0.10	3.06 ± 0.11	3.05 ± 0.11	3.02 ± 0.13^*^
Frontal	3.04 ± 0.15^*^	3.08 ± 0.12	3.11 ± 0.11	3.10 ± 0.11	3.10 ± 0.12	3.10 ± 0.11	3.06 ± 0.14^*^
Temporal	3.17 ± 0.17	3.20 ± 0.16	3.22 ± 0.15	3.22 ± 0.16	3.20 ± 0.18	3.22 ± 0.15	3.18 ± 0.18^*^
Parietal	2.84 ± 0.15^*^	2.89 ± 0.14^*^	2.92 ± 0.13	2.92 ± 0.14	2.93 ± 0.16	2.91 ± 0.14	2.89 ± 0.16
Occipital	2.64 ± 0.17^*^	2.68 ± 0.13	2.71 ± 0.12	2.70 ± 0.12	2.70 ± 0.12	2.70 ± 0.14	2.66 ± 0.13^*^

eGFR, estimated glomerular filtration rate; UACR, urine albumin to creatinine ratio; elevated UACR if the value was ≥17 mg/g for men and ≥25 mg/g for women.

SBP, systolic blood pressure; DBP, diastolic blood pressure; WMH, whitematter hyperintensities; ICV, intracranial volume.

Values are expressed as mean (± standard deviation) for continuous variables or N (%) for categorical variables.

^*^p < 0.05 compared to the reference group (eGFR 90-104 or normal UACR) after Bonferroni correction.

**Table 2:**
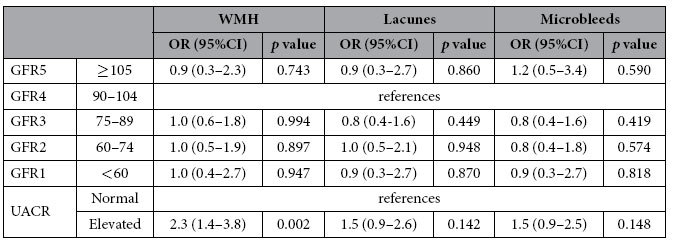
Kidney function and cerebrovascular disease markers (WMH, lacunes and microbleeds).

Logistic regression analysis adjusted for age, sex, history of hypertension, diabetes mellitus, hyperlipidemia, ischemic heart disease, and stroke, fasting glucose, systolic blood pressure, diastolic blood pressure, total cholesterol level, body mass index, status of current smoking and alcohol drinking, education level, intracranial volume, and GFR groups or UACR. GFR, glomerular filtration rate (ml/min/1.73 m^2^); UACR, urine albumin to creatinine ratio; elevated UACR if the value was ≥17 mg/g for men and ≥25 mg/g for women. WMH, whitematter hyperintensities; dichotomized into absent to minimal or moderate to severe WMH. Lacunes and microbleeds were dichotomized into categories of absent or present.

**Table 3:** The effect of eGFR or albuminuria on regional cortical thickness.

	Overall		Frontal lobe		Temporal lobe		Parietal lobe		Occipital lobe	
	B (SE)	*p*value	B (SE)	*p*value	B (SE)	*p*value	B (SE)	*p*value	B (SE)	*p*value
eGFR (ml/min/1.73m^2^)										
≥105	−0.005 (0.012)	0.678	−0.006 (0.013)	0.659	−0.023 (0.018)	0.770	0.001 (0.015)	0.953	−0.003 (0.014)	0.802
90–104	references									
75–89	0.008 (0.007)	0.278	0.010 (0.008)	0.174	0.011 (0.011)	0.758	0.001 (0.009)	0.879	0.013 (0.008)	0.125
60–74	−0.002 (0.008)	0.800	0.001 (0.009)	0.939	0.004 (0.013)	0.305	−0.011 (0.011)	0.303	0.010 (0.010)	0.289
<60	−0.013 (0.014)	0.341	−0.009 (0.015)	0.543	0.006 (0.021)	0.203	−0.024 (0.018)	0.167	−0.008 (0.016)	0.600
UACR (mg/g)										
Normal	references									
Elevated	−0.023 (0.009)	0.015	−0.027 (0.010)	0.008	−0.024 (0.015)	0.100	−0.010 (0.012)	0.418	−0.031 (0.011)	0.005

eGFR, estimated glomerular filtration rate; UACR, urinary albumine to creatinine ratio; elevated UACR if the value was ≥30 mg/g; SE, standard error. Linear regression analysis adjusted for age, sex, history of hypertension, diabetes mellitus, hyperlipidemia, ischemic heart disease, and stroke, fasting glucose, systolic blood pressure, diastolic blood pressure, total cholesterol level, body mass index, status of current smoking and alcohol drinking, education level and intracranial volume, GFR groups or UACR.
